# New approach for combined aortic valve and coronary procedures through the left anterior minithoracotomy

**DOI:** 10.1093/icvts/ivad214

**Published:** 2024-01-04

**Authors:** Oleksandr Babliak, Volodymyr Demianenko, Dmytro Babliak, Anton Marchenko, Yevhenii Melnyk, Oleksii Stohov

**Affiliations:** Diagnostic and Treatment Center for Children and Adults of the Dobrobut Medical Network, Kyiv, Ukraine; Diagnostic and Treatment Center for Children and Adults of the Dobrobut Medical Network, Kyiv, Ukraine; Diagnostic and Treatment Center for Children and Adults of the Dobrobut Medical Network, Kyiv, Ukraine; Diagnostic and Treatment Center for Children and Adults of the Dobrobut Medical Network, Kyiv, Ukraine; Diagnostic and Treatment Center for Children and Adults of the Dobrobut Medical Network, Kyiv, Ukraine; Diagnostic and Treatment Center for Children and Adults of the Dobrobut Medical Network, Kyiv, Ukraine

**Keywords:** Minimally invasive cardiac surgery, Coronary artery disease, Aortic valve replacement, Total coronary revascularization via left anterior thoracotomy, Total coronary revascularization via left anterior thoracotomy, Coronary artery bypass grafting

## Abstract

**OBJECTIVES:**

We have developed a novel technique for accessing the aortic valve (AoV) through the left anterior minithoracotomy (LAmT). This approach has been used in patients requiring both AoV surgery and coronary artery bypass grafting (CABG).

**METHODS:**

From April 2023 to July 2023, we performed 6 concomitant AoV procedures and CABG through the LAmT. The mean age was 71.5 [standard deviation (SD): 5.8; 64; 82] years, and the mean left ventricular ejection fraction was 53% (SD: 12.1; 30; 60). Surgical technique includes LAmT in the fourth intercostal space, peripheral cardiopulmonary bypass, aortic cross-clamping using transthoracic clamp, cold blood cardioplegia, conventional oblique aortotomy and special surgical exposure manoeuvres, aimed to position the ascending aorta and AoV close to the surgical incision.

**RESULTS:**

AoV was effectively visualized and the procedure was performed as planned in all 6 patients. No conversion to sternotomy was required. AoV replacement with biological prosthesis was performed in 6 (100%) patients. Conventional surgical instruments were used in all cases. The long-shafted instruments were not required. Knot-pusher was used in 4 (67%)cases. Concomitant complete revascularization was achieved in all cases. The mean number of distal anastomosis was 2.0 (SD: 0.6; 1; 3). Total operation time was 371 (SD: 43; 300; 420) min, cardiopulmonary bypass time was 253 (SD: 36; 193; 284) min and cross-clamp time was - 162 (SD: 29; 128; 214) min. intensive care unit stay was—1.5 (SD: 0.55; 1; 2) days, total hospital stay was—7.3 (SD: 1; 6; 9) days. There were no revisions for bleeding, no strokes or other major complications, and no hospital or 30-days mortality.

**CONCLUSIONS:**

The simultaneous performance of AoV replacement and multivessel CABG through a single left anterior thoracotomy is technically feasible and can be carried out by experienced surgeons. However, a larger number of cases are required to fully comprehend the potential limitations of this procedure.

**Figure:**
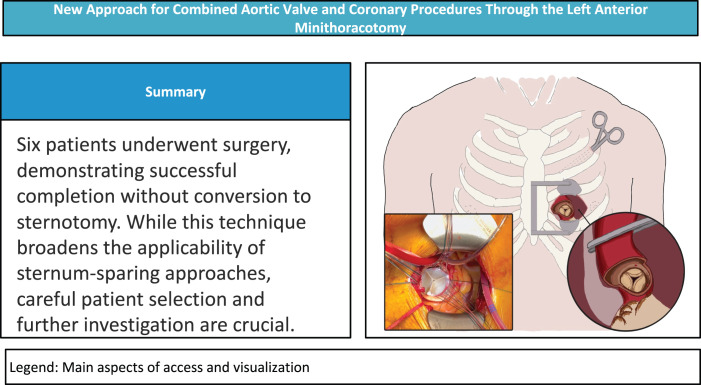

## INTRODUCTION

Aortic valve (AoV) surgery traditionally involved a sternotomy. The advancements in minimally invasive surgical techniques made possible that most of the isolated AoV surgery can be safely and effectively performed through the right minithoracotomy approach [1, 2]. However, combined procedures including aortic valve replacement (AVR) and coronary artery bypass grafting (CABG) are still performed through the median sternotomy.

Recently, we discovered that MVR can be effectively exposed through the left anterior minithoracotomy (LAmT) [3, 4], which we routinely use as a first-choice approach for multivessel CABG patients [5–7]. The possibility to access the AoV through the LAmT made it possible to perform combined coronary with AoV surgical procedures in selective patients through the single minithoracotomy.

The objective of this article is to analyse a novel technique for accessing the AoV through the LAmT in patients who need both AoV and coronary surgery.

## MATERIALS AND METHODS

### Study population

This study involves a retrospective analysis of a cohort of patients from a single centre. It includes all patients who underwent both AoV surgery and coronary revascularization simultaneously through a single LAmT. These procedures were conducted between April and July 2023, using cardiopulmonary bypass (CPB) and cardioplegic cardiac arrest. The study encompassed all patients operated with this approach, totalling 6 patients. Aortic stenosis was the predominant valve lesion in all patients.

In the same time frame, two patients with both severe AoV stenosis and multivessel coronary disease underwent surgery via sternotomy. This decision was made based on the preoperative computed tomography (CT) scan results that revealed a calcified ascending aorta. Consequently, these patients were not included in the study.

### Statistical analyses

Data were presented as mean (standard deviation; minimum; maximum) or number (percentage). Normality of all the continuous variables was investigated and no skew in distribution of data was observed. No missing data were observed.

### Ethical standards

This study was approved by the institutional review board of the Diagnostic and Treatment Centre for Children and Adults of the Dobrobut Medical Network [protocol approval number: 16 (08.12.21)]. Individual informed consent was waived due to the retrospective nature of the study.

### Preoperative evaluation

In addition to routine preoperative assessments, all patients underwent a CT scan (Siemens Somatom X.cite (Siemens Healthineers, Erlangen, Germany); 128 slices of 0.5 mm size) to screen for atherosclerotic disease and anatomical abnormalities in the aorta and major arterial branches. This was crucial for planning the CPB strategy, selecting the appropriate peripheral arterial cannulation site and ensuring safe aortic cross-clamping.

**Figure 1:**
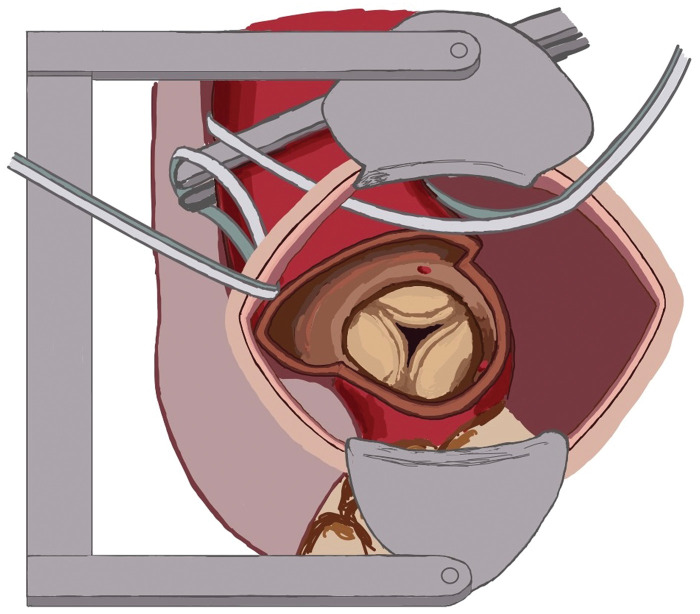
Schematic picture of AoV exposure. Tapes are used for displacement of the heart to the left pleural space. Thus, AoV posised in the middle of left anterior thoracotomy. AoV: aortic valve.

Furthermore, special attention was given to the detection of maximal CT distance from the posterior portion of AoV annulus (base of noncoronary cusp) to the skin level (Fig. 1). We perform that to estimate the expected deepness of AoV location. This distance was measured on transverse CT planes preoperatively.

### Anaesthesia

The anaesthesia approach utilized a low-dose opioid and a multimodal strategy for both the initiation and continuation of anaesthesia. All patients were intubated with a single-lumen endotracheal tube and a 9-Fr Arndt bronchial blocker in the left main bronchus, which is our preferred technique for single-lung ventilation. A jugular vein cannula (DLP femoral arterial cannula 17 Fr, Medtronic, Minneapolis, MN, USA) was inserted preoperatively by anaesthesiologist at the time of the central line insertion, and a 5000 unit dose of heparin was administered to prevent clotting.

Transoesophageal echocardiography was used in all patients.

### Surgical technique

The technique for isolated multivessel CABG through the LAmT, known as total coronary revascularization via left anterior thoracotomy (TCRAT)-CABG technique, has been described in detail by our team in publications [6–8]. In this paper we present a detailed description of the technique used for combined procedures—CABG and AVR through the LAmT.

All patients are placed in a supine position with an inflatable pad under the left chest. After administering heparin at a dose of 300 U/kg, CPB lines were inserted. For arterial inflow an EOPA arterial cannula with a size of 18–20 Fr (Medtronic) was used. For venous return, a Bio-Medicus multi-stage femoral venous cannula with a size of 25 Fr (Medtronic) was used.

To access the heart and left internal mammary artery (LIMA) during the TCRAT-CABG procedure, a small skin incision measuring 7–8 cm was made anteriorly along the 4th intercostal space in males or under the breast in women, starting from the left sternal edge. The subcutaneous tissue was then mobilized and the pectoral muscle was split along its fibres. The chest was entered through the 4th intercostal space without rib resection and a rib spreader retractor with concave moveable blades (Babliak RETRACTOR, Idol Company, Antalya, Turkey) was then inserted.

LIMA harvesting was done with a skeletonized or semiskeletonized technique using LIMA harvesting retractor (Babliak IMA Lifting System, Idol Company, Antalya, Turkey), long conventional surgical instruments, diathermy 15 cm tip extension and single-lung ventilation. CPB was initiated at the end of LIMA harvesting to facilitate exposure of the proximal portions of the conduit. Vacuum-assisted venous return was routinely used during CPB.

The pericardium was opened in a T-shaped manner from the apex to the ascending aorta. The ascending aorta was then encircled with a tape. Aorta was cross-clamped with a transthoracic aortic clamp (Babliak Aortic Clamp, 80-mm right curved 2X3 DeBakey jaw, locked, 230 mm working length, Idol Company, Antalya, Turkey) that was inserted through the second intercostal space between the midclavicular and anterior axillary lines, and cold blood antegrade intermittent cardioplegia was administered. Cardioplegia was readministered every 15–25 min in the root during coronary grafting or directly to the coronary ostia during AVR.

All coronary anastomoses were performed with conventional instruments and techniques. Special manoeuvres were used to facilitate the exposure of distal coronary targets.

Regarding surgical sequence, all distal anastomoses to the lateral and inferior wall targets were done before AVR, then AVR was performed, and LIMA to left anterior descending artery anastomosis was done after AVR.

AoV was accessed through the conventional oblique aortotomy. Special manoeuvres were performed to expose the ascending aorta and AoV closer to the surgical incision. First, left-sided pericardial stay sutures were completely released, which helps the apex of the heart to slide into the left pleural space. Second, the tape encircling the edges of the transthoracic aortic clamp was led out through the operation wound and pulled to the right, which moved the ascending aorta to the middle of the operation field (Fig. 1). Once the aorta was opened two or three commissural stitches were placed and pulled out to further improve the AoV exposure. A narrow spatula or wall sucker was used for aortic wall retraction during aortic annulus debridement, stitches placement and tying. Conventional instruments were used for stitches placement. Knot-pusher was used in 4 of 6 cases to facilitate knot tying. AVR and aortotomy can be performed from either side of the surgical table. In our series, we conducted these procedures in 4 patients (67%) from the right side and in 2 patients (33.3%) from the left side. Left ventricle was vented through the left atrium appendage and additionally directly through the AoV.

Finally, the LIMA to left anterior descending artery anastomosis was carried out and aorta was unclamped.

Proximal anastomosis was performed on the beating heart using a conventional side-biting clamp in 3 (50%) cases, during a single aortic cross-clamp—2 (33.3%) cases. In one case, no proximal anastomoses were required.

CPB weaning was performed with double lung ventilation. Transoesophageal echocardiography was performed after the CPB weaning and AoV prosthesis was evaluated.

A single drainage tube (preferably a BLAKE™ Silicone Drain with a round hubless design, 24 Fr in size) was placed through the second intercostal space at the site where the transthoracic aortic clamp had been applied.

At the end of the procedure, single-lung ventilation was restarted to aid in surgical hemostasis. The detailed technical aspects are outlined in Video 1.

## RESULTS

**Figure 2:**
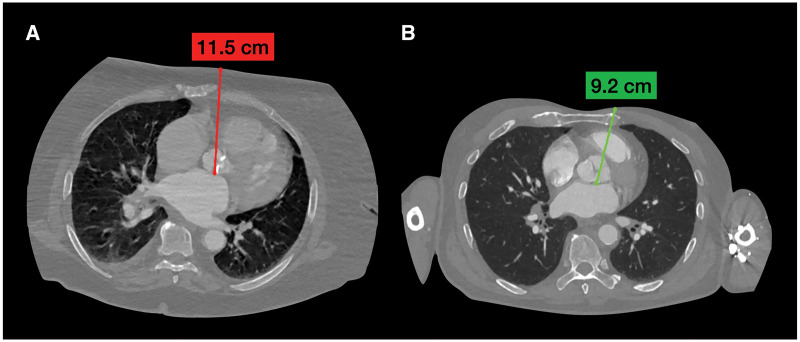
Computed tomography distance from the posterior aortic valve annulus to the skin level. (**A**) Knot-pusher was used during aortic valve replacement. (**B**) Knot-pusher was not used.

The surgical procedure described above was successfully completed in all six patients, and there was no need to convert to a sternotomy during the surgeries. All patients underwent AVR with The Carpentier–Edwards Perimount Magna Ease aortic valve (Edwards Lifesciences, Irvine, CA) with a diameter of 19 mm in 1 (16.6%) case, 21 mm—2 (33.4%) cases and 23 mm—3 (50%) cases. CT distance from the skin level to AoV posterior annulus was 10.92 cm [standard deviation (SD): 1.35; 9.2; 12.7]. A knot-pusher was used in 4 (67%) cases for tying knots in patients with CT distance of >10 cm, usually for the sutures that were placed to the noncoronary part of the aortic annulus (Fig. 2).

In this series, noteworthy prevalence of multivessel disease was observed, with one patient exhibiting triple-vessel disease and four patients (67%) manifesting double-vessel disease (Table 1). All patients achieved concomitant complete revascularization with a mean number of distal anastomoses of 2.0 (SD: 0.6; 1; 3). Regarding conduits, LIMA and saphenous vein grafts were used in 6 (100%) and 5 (83.4%) patients, respectively. Intraoperative TEE confirmed adequate valve function and no paravalvular leak. Intraoperative parameters are presented in Table 2.

**Table 1:** Preoperative characteristics

Characteristic	Value
Age (years)	71.5 (SD: 5.8; 64; 82)
Female	4 (66.6)
Arterial hypertension	5 (83.3)
BMI >30	2 (33.3)
Diabetes mellitus	4 (66.6)
Ejection fraction (%)	53 (SD: 12.1; 30; 60).
Severe aortic stenosis	4 (66.6)
Aortic valve mean gradient (mmHg)	48.7 (SD: 15; 30; 71)
No aortic valve insufficiency	2 (33.3)
Mild aortic valve insufficiency	4 (66.6)
Triple-vessel disease	1 (16.6)
Double-vessel disease	4 (66.6)
Atrial fibrillation	3 (50)
EuroSCORE II	3.18 (SD: 2; 1.31; 6.29)
CT distance from skin level to AoV posterior annulus (cm)	10.92 (SD: 1.35; 9.2; 12.7)

Data are presented as mean (SD; minimum; maximum) or *n* (%).

AoV: aortic valve; BMI: body mass index; CT: computed tomography; SD: standard deviation.

**Table 2:** Intraoperative data

Operative time (min)	371 (SD: 43; 300; 420)
CPB time (min)	253 (SD: 36; 193; 284)
Cross-clamp time (min)	162 (SD: 29; 128; 214)
Distal anastomoses	2.0 (SD: 0.6; 1; 3)
Conversion	0

Data are presented as mean (SD; minimum; maximum) or *n* (%).

CPB: cardiopulmonary bypass; SD: standard deviation.

The mean postoperative intensive care unit and hospital length of stay were 1.5 (SD: 0.55; 1; 2) and 7.3 (SD: 1; 6; 9) days, respectively. There was no incidence of pleural effusion, no AV block and no temporary pacemaker requirement, no pericarditis, perioperative stroke or myocardial infarction (newly developed signs of myocardial ischaemia based on the Fourth Universal Definition of Myocardial Infarction criteria [8]). Two cases (33.3%) of postoperative atrial fibrillation were observed. There were no cases of revision for bleeding, and no hospital or 30-day mortality (Table 3).

**Table 3:** Early postoperative outcomes

Mechanical ventilation time (min)	386 (SD: 183; 240; 750)
Cumulative chest tube drainage 12 h (ml)	285 (SD: 82; 180; 400)
ICU length of stay (days)	1.5 (SD: 0.55; 1; 2)
Hospital length of stay (days)	7.3 (SD: 1; 6; 9)
Temporary pacemaker requirement	0
Postoperative atrial fibrillation	2 (33.3)
Pericarditis	0
Pleural effusion aspirated	0
Stroke or transient cerebral ischaemic attack	0
Myocardial infarction	0
30-Day mortality	0

Data are presented as mean (SD; minimum; maximum) or *n* (%).

ICU: intensive care unit; SD: standard deviation.

## DISCUSSION

The main advantage of the sternum-sparing approach is to minimize surgical trauma and eliminate the risk of osteomyelitis and deep sternal wound infection [1, 2]. The primary finding presented in this paper is that AoV can be effectively reached via the LAmT approach, allowing for adequate AoV exposure required for prosthesis implantation. This achievement enables the feasibility of performing both AoV and coronary surgery simultaneously through a single minithoracotomy.

Previous reports about combined AoV and CABG surgery with sternum-sparing techniques included different strategies. Bilateral thoracotomies have been described by Smit *et al.* [9]. The combination of upper hemisternotomy and left thoracotomy was described by Sellin *et al.* [10]. The combination of percutaneous coronary stent angioplasty with AVR via the right minithoracotomy has been documented, as well as the concurrent performance of coronary revascularization on the beating heart through the left anterolateral thoracotomy, followed by Transcatheter Aortic Valve Implantation, as reported in references [11, 12]. The main disadvantage of these strategies is a high selection of patients because of surgical and interventional procedural limitations.

The average mean aortic cross-clamp time in our study was 162 min (SD: 29; 128; 214). This duration should be carefully considered, and preoperative planning for myocardial protection should be undertaken accordingly. Given that extended ischaemic times are inevitable in cases involving both valve and coronary procedures, our preferred approach is the use of multi-dose cold blood cardioplegia.

Our objective was to decrease the high selectivity associated with the utilization of the sternum-sparing approach for combined AVR and CABG surgery. As a result, 6 out of our 8 consecutive patients (75%) undergoing combined AVR and CABG surgery underwent the procedure through a single minithoracotomy, while 2 patients (25%) underwent the surgery via a sternotomy.

This study encompasses all patients who underwent combined AVR and coronary procedures, starting with the first patient operated through a single left minithoracotomy. Initially, we utilized a bilateral thoracotomies approach for these patients and used this technique in 12 cases between 2020 and 2022. We found this approach feasible but time-consuming and technically complex, as the surgeon's attention had to be divided between two incisions. With the development of the technique described in this article, the single left minithoracotomy approach has become our preferred method for performing simultaneous AVR and CABG surgeries.

Nevertheless, for isolated AVR, our preferred approach is the right anterior minithoracotomy in the second intercostal space.

Several factors can influence the quality of AoV exposure through the LAmT, but these factors were not examined in this initial study. The cardiac surgeon's experience with routine TCRAT-CABG procedures through the left anterior thoracotomy appears to be an important factor in facilitating the initiation of combined procedures using the same incision.

Furthermore, patients with atherosclerotic ascending aorta and those who have previously undergone open-heart surgery are not suitable candidates for the technique outlined in this article.

Regarding the additional advantages of this approach, aside from avoiding sternotomy, we can preliminarily suggest that postoperative recovery is comparable to that of other patients who undergo cardiac surgery via a small right or left thoracotomy. However, further research is needed to assess patient satisfaction comprehensively.

## CONCLUSION

Overall, our findings indicate that the novel approach to AoV surgery through the LAmT presents a viable alternative to the traditional sternotomy for simultaneous AVR and CABG. Nevertheless, it may not be suitable for all patients, and larger studies with more extensive patient cohorts are essential to validate the reliability and efficacy of this approach.

## Data Availability

The data underlying this article cannot be shared publicly because of the policy for internal databases of the Diagnostic and Treatment Centre for Children and Adults of the Dobrobut Medical Network. The data could be shared partially on reasonable request to the corresponding author.

## Notes

Presented at the 37th EACTS Annual Meeting, Vienna, Austria, 4–7 October 2023.

## ABBREVIATIONS

AoV: Aortic valve
AVR: Aortic valve replacement
CABG: Coronary artery bypass grafting
CPB: cardiopulmonary bypass
CT: Computed tomography
LAmT: Left anterior minithoracotomy
LIMA: Left internal mammary artery
SD: Standard deviation
TCRAT: Total coronary revascularization via left anterior thoracotomy
